# Intermedin Suppresses Pressure Overload Cardiac Hypertrophy through Activation of Autophagy

**DOI:** 10.1371/journal.pone.0064757

**Published:** 2013-05-29

**Authors:** HuaLi Chen, Xue Wang, MingMing Tong, Dan Wu, Sisi Wu, JiaXiang Chen, XiaoXiao Wang, XuLei Wang, Yu Kang, Hong Tang, ChaoShu Tang, Wei Jiang

**Affiliations:** 1 Molecular Medicine Research Center, State Key Laboratory of Biotherapy, West China Hospital, Sichuan University, Chengdu, Sichuan, P.R. China; 2 School of Life Sciences and Bioengineering, Southwest Jiaotong University, Chengdu, Sichuan, P.R. China; 3 Department of Cardiology, West China Hospital, Sichuan University, Chengdu, Sichuan, P.R. China; 4 Teaching Hospital of Chengdu University of Traditional Chinese Medicine, Chengdu, Sichuan, P.R. China; 5 Department of Physiology, Peking University Health Science Center, Beijing, P.R. China; Loyola University Chicago, United States of America

## Abstract

Left ventricular hypertrophy is a maladaptive response to pressure overload and an important risk factor for heart failure. Intermedin (IMD), a multi-functional peptide, plays important roles in cardiovascular protection. In this study, we revealed an autophagy-dependent mechanism involved in IMD’s protection against cardiac remodeling and cardiomyocyte death in heart hypertrophy. We observed that transverse aortic contraction (TAC) induction, Ang II or ISO exposure induced remarkable increase in the expression of endogenous IMD and its receptor components, CRLR, RAMP1 and RAMP3, in mouse hearts and H9c2 cell cultures, respectively. Furthermore, the heart size, heart weight/body weight ratios, cardiomyocyte size and apoptosis, interstitial collagen, hypertrophic markers including ANP and BNP expression were also significantly increased, which were effectively suppressed by IMD supplementation. In addition, IMD induced capillary angiogenesis and improved functions in hypertrophic hearts. We further observed that IMD induced strong autophagy in hypertrophic hearts and cultured cells, which was paralleling with the decrease in cardiomyocyte size and apoptosis. Furthermore, an autophagy inhibitor, 3-MA, was used to block the IMD-augmented autophagy level, and then the protection of IMD on cardiomyocyte hypertrophy and apoptosis was almost abrogated. We also observed that IMD supplementation stirred intracellular cAMP production, and augmented the ERK1/2 phosphorylation induced by Ang II/ISO exposure in H9c2 cells. In addition, we inhibited PI3K, PKA and MAPK/ERK1/2 signaling pathways by using wortamannin, H89 and PD98059, respectively, in H9c2 cells co-incubating with both IMD and Ang II or ISO, and observed that these inhibitors effectively reduced IMD-augmented autophagy level, but only H89 and PD98059 pre-incubation abrogated the anti-apoptotic action of IMD. These results indicate that the endogenous IMD and its receptor complexes are induced in hypertrophic cardiomyocytes and proposed to play an important role in the pathogenesis of cardiac hypertrophy, and the autophagy stirred by IMD supplementation is involved in its protection against cardiomyocyte hypertrophy and apoptosis through the activation of both cAMP/PKA and MAPK/ERK1/2 pathways.

## Introduction

Cardiac hypertrophy, a major predictor for the development of coronary artery disease and heart failure, develops in response to hemodynamic overload [1]. Cardiac hypertrophy is characterized as an abnormal enlargement of the heart muscle originating from an increase in cell size of myocytes and proliferation of non-muscle cells [1], [2]. Biochemically, the hypertrophic response is abnormalities in contractile protein content and myofilament organization, and re-expression of embryonic markers [3]. At the cellular and molecular level the hypertrophic adaptation transition is a complex process that local autocrine/paracrine network imbalance and circulating biologically active mediators are involved in the heart’s molecular response to increased wall stress and the development of hypertrophy [4]. Mounting evidence supports that autocrine and paracrine mechanisms, mediated by factors released by the resident cardiac cells, play an essential role in the remodeling process of the hypertrophic hearts [5]. Therefore, identification of specific autocrine/paracrine cell-derived factors that improve cardiac function has been intensively studied as potential pharmacologic targets to prevent and reverse cardiac hypertrophic diseases [4]–[6]. Intermedin (IMD), also called adrenomedullin (ADM) 2, is a novel member of the calcitonin/calcitonin gene-related peptide (CGRP) family [7]. It was proven to be a multifunctional peptide with cardiovascular homeostasis regulation similar to another CGRP family member, ADM, by potently dilating systemic and pulmonary vessels, influencing the regional distribution of blood flow, inducing cardiac contractility, and increasing urinary flow and urinary sodium excretion [8]. Like the other CGRP family members, IMD also binds to the calcitonin receptor-like receptor (CRLR), a type II G protein-coupled receptor, whose ligand binding specificity is dictated by three subtypes of receptor activity-modifying proteins (RAMPs) [7], [8]. IMD is a selective agonist for CRLR/RAMP1 and CRLR/RAMP3 receptor complexes [9]. The distribution of IMD and its receptors in the cardiovascular tissues is compatible with a physiological role in the regulation of peripheral circulation [8]. IMD has an interesting character of the robust expression in the diseased cardiovascular system, which indicates an important role in cardiovascular pathophysiology [8]. Several studies have reported that the endogenous IMD in cardiomyocytes was significantly increased in response to hypertrophic stimuli [8], and IMD supplementation antagonized cardiomyocyte hypertrophy by cAMP/PKA activation [10]. However, cardiac hypertrophy is controlled by a complex signal transduction and gene regulatory network [1]–[3]. Recently, autophagy, a dynamic process involving the bulk degradation of cytoplasmic organelles and proteins, has been proven to be involved in the pathogenesis of cardiac hypertrophy [11]. Now, little is known about the regulation of IMD on autophagy in hypertrophic process [7], [8].

It is likely that the basal level of constitutive autophagy in the heart is a homeostatic mechanism and is protective [12]. It is also important for maintaining the amino acid pool within cardiomyocytes and the entire heart, and degrades large amount of cellular protein under starvation conditions [11], [12]. The main features of cardiac hypertrophy are increased cell size, enhanced protein synthesis, and heightened organization of the sarcomere, and associating with the aggregation of misfolded proteins and damage of sub-cellular organelles including mitochondria, which can be cleared by a bulk degradation autophagic process [1], [11]. However, autophagy appears to play a complex role in cardiac hypertrophy [11]. Induction of ventricular hypertrophy is usually associated with decreased autophagy, which facilitates the accumulation of unfolded proteins and worsens cardiac functions, whereas autophagy is enhanced during the regression of hypertrophy [13]. Hurtado and colleagues [14] observed a significant increase in Beclin-1, an essential component for autophagic process, in the peri-infarct area of ADM-deficient mouse brain, while, the effect of IMD on autophagy is still unknown [7], [8]. Here, we report that IMD attenuates the cardiac hypertrophy, fibrosis and myocyte apoptosis in a mouse model of myocardial hypertrophy induced by transverse aortic coarctation (TAC) and H9c2 cells stimulated by angiotensin II (Ang II) or isoprenaline (ISO) through inducing the protective autophagy, and further improving cardiac performance.

## Materials and Methods

### Materials

DL-Isoproterenol hydrochloride (ISO), Wortmannin, AngiotensinII (Ang II), Dimethyl sulfox-ide (DMSO), 3-methyladenine (3-MA), PD98059, wortmannin and phalloidine from Sigma-Aldrich Chemicals (St Louis, MO, USA); H89 from Millipore Corporation (MA, USA); Alexa Fluor 568-conjugated phalloidin from Invitrogen (USA); TRIzol® Reagent, and lipofectamin^TM^2000 from Invitrogen (Carlsbad, CA, USA); dNTP from Takara Bio Inc (Shiga, Japan); SuperScript™ II reverse transcriptase from Promega; iQ™ SYBR® Green Supermix from Bio-Rad Laboratories, Inc. (Hercules, CA, USA); Oligo (dT) 15 primer from Promega; specific rabbit polyclonal antibody against mouse and rat IMD from Phoenix Pharmaceutical (Belmont, CA, USA); rabbit polyclonal anti-von Willebrand Factor (vWF) antibody (sc-14014) from Santa Cruz Biotechnology (CA, U.S.A.); Akt and phosphor-Akt (Thr473), ERK and phosphor-ERK (Thr202/Tyr204), Anti-caspase3, anti-LC3B and anti-β-actin antibodies from Cell Signaling Technologies (Beverly, MA, USA), 4′6′-diamidino-2-phenylindole (DAPI) and terminal deoxynucleotidyl transferase dUTP nick end labeling (TUNEL) histochemical fluorescent detection kit from Roche (Roche Applied Science, Indianapolis, IN, USA). cAMP assay kit from R&D Systems (R&D Systems, MN, USA). All pairs of PCR primers were synthesized by Shenggong Biotechnology (Shanghai, China). Other chemicals and reagents were of analytical grade.

### Peptide Synthesis

Mouse IMD peptide (IMD8–47) with the sequence Val-Gly-Cys-Val-Leu-Gly-Thr-Cys-Gln-Val-Gln-Asn-Leu-Ser-His-Arg-Leu-Trp-Gln-Leu-Val-Arg-Pro-Ala-Gly-Arg-Arg-Asp-Ser-Ala-Pro-Val-Asp-Pro-Ser-Ser-Pro-His-Ser-Tyr-NH_2_ with a intramolecular disulfide bond between Cys3-Cys8 [15] was synthesized by ShineGene Bio-Technologies (Shanghai, China) through the solid-phase fluorenylmethoxycarbonyl method with a simultaneous multiple solid-phase peptide synthesizer (PSSM 8 system, Shimadzu, Tokyo, Japan) and analyzed by reverse phase HPLC (Agilent 1050, Agilent Technologies, Santa Clara, CA, USA) with a Cromasil C18 column and mass spectrometry using a Finnigan LCQ (Thermo/Finnigan Ltd., Waltham, MA, USA).

### Animals

Male C57BL/6 mice (animal center, Health Sciences Center, Sichuan University), 8–9 weeks old, were housed under standard conditions (room temperature 20±1°C, humidity 60±10%, lights from AM 6∶00 to PM 18∶00) and given free access to standard rodent chow and water. All experimental procedures were performed in compliance with the Guide for the Care and Use of Laboratory Animals published by the US National Institutes of Health (NIH publication No.85–23, Revised 1985) and approved by the Animal Care and Use Committee of Sichuan University. Mice, at 8 weeks old, were anesthetized with sodium pentobarbital (45 mg/kg intraperitoneally), then intubated and ventilated with a rodent ventilator (Harvard Apparatus, Holliston, MA, USA). The chest was opened, and following blunt dissection through the intercostals muscles, the thoracic aorta was identified. A 7–0 silk suture was placed around the transverse aorta and tied with a 6–0 silk suture against a blunt needle (26 gauge), which was subsequently removed [16]. IMD-treated animal was delivered subcutaneously with IMD (200 ng/kg/hour) in normal saline by a mini-osmotic pump for 4 weeks. Sham-operated mice (control group) underwent a similar surgical procedure without constriction of the aorta, and administrated subcutaneously with 4-week normal saline by a mini-osmotic pump (ALZET model 2004 osmotic pump; ALZA Corp., CA, USA). After 4 weeks, surviving mice were subjected to transthoracic echocardiography and cardiac catheterization to determine heart rate and proximal aortic pressure.

### Echocardiography and Cardiac Haemodynamic Measurements

Transthoracic echocardiography was performed with a GE Vivid 7 (GE Health Medical, Milwaukee, WI, USA) equipped with a 12-MHz imaging transducer. All recordings were performed in animals under light anesthesia (45 mg/kg pentobarbital sodium i.p.) and positioned on a heated pad in a recumbent position. Measurements were performed at the midpapillary level from well aligned M-mode images from the parasternal short-axis view [17]. All echocardiography was performed by the same investigator who was blinded to the treatments. After the echocardiography, the animals were mechanically ventilated with the chest opened, and then left ventricular (LV) performance was measured by inserting a Pressure-Volume catheter 1.2 F transducer (4.5 mm electrode spacing, Serial No. 112B-B057, SCIsense Inc., Ontario, Canada) into the left ventricle from apex, and positioned along the cardiac longitudinal axis, with temperature maintained at 36–38°C by use of the heated pad [17]. The signals were recorded by an eight-channel physiological recorder (iWorx 308, iWorx/CB Sciences, Inc., Dover, NH, USA). The maximal slope of systolic pressure increment (dP/dt_max_) and diastolic pressure decrement (dP/dt_min_) were calculated on the base of the left ventricular systolic and end-diastolic pressures. After haemodynamic recording, the mice were euthanized by pentobarbital overdose (i.p.), and the hearts were removed and divided into three parts for RNA, protein extracted and histological study.

### Histological Study

The heart tissue sections were paraffin-embedded, and cut into 4 µM serial sections, and then stained with hematoxylin and eosin (H&E), Masson trichrome (Baso Biological technology Inc., Zhuhai, China), TUNEL, vWF or IMD antibody, a Nikon system (Nikon, Japan) was used to capture digital images. Cardiomyocyte size was assessed on H&E-stained sections. About 100–150 randomly chosen cardiomyocytes from each group (n = 3–4) were analyzed by use of Image J software to measure cross-sectional cardiomyocyte area and presented as µm^2^ [18]. The amount of myocardial interstitial fibrosis was evaluated by Image-Pro Plus software (Media Cybernetics, USA) on Masson’s trichrome stained sections. About 30–50 randomly chosen frames from each group (n = 3–4) were assayed [18]. Apoptosis in cardiomyocytes was determined following reagent manufacturers’ instructions, and then high-power fields (12 to 20 at ×400 magnifications) were measured to count the number of TUNEL-positive cardiomyocyte nuclei in the control, TAC and IMD-treated hearts, respectively. Only nuclei that were clearly purplish red were scored. The percentage of TUNEL-positive cells was calculated as the ratio between TUNEL-positive and DAPI-stained nuclei×100 [17]. The immunohistochemical localization of IMD was studied according to the method described earlier [17]. After the sections were treated with 3% H_2_O_2_ to block endogenous peroxidase, they were first incubated for 1 h at room temperature with rabbit polyclonal anti-IMD antibody at a dilution of 1∶150 or anti-vWF antibody at 1∶400 dilution, and then with biotinylated goat IgG (Vector Laboratories, Burlingame, CA, USA). IMD or vWF immunoreactivity was visualized by use of diaminobenzidine substrate. The IMD-like immunoreactivity was indicated by a brown color in the cytoplasm, and the spots of vWF positive immunoreactivity indicated the vessel endothelial cells. ImagePro-Plus 5.0 (Media Cybernetics, Silver Spring, MD, USA) was used to analyze the mean optical density of IMD staining, and count capillary density, which was assessed light-microscopically on vWF antibody-stained cardiac tissue by analyzing 10 random fields (at ×200 magnifications) per section (n = 4/group), the results were presented as capillaries/mm^2^ [19]. Any immunostained endothelial cell or cell cluster was considered as a single microvessel. In addition, the reactivities of the antibody against IMD protein from mice, rats and humans were 100%. No cross-reactivity was found with any CGRP and ADM.

### Hypertrophic H9c2 Cell Models Induced by Ang II and ISO Insults

The H9c2 cell line is a subclone of the original clonal cell line derived from embryonic BD1X rat heart tissue, which holds many cardiomyocyte characteristics [20]. All cells we used were passaged from an initial CRL-1446 cell culture of the American Type Culture Collection (CRL-1446; ATCC, Rockville, MD). Cells were cultured in Dulbecco’s modified Eagle’s medium with 10% fetal bovine serum, 100 U/mL penicillin, and 100 mg/mL streptomycin, at 37°C in a humidified incubator containing 95% air and 5% CO_2_. The media was refreshed every 3 days. Cells cultured at about 80% confluence were treated with either Ang II(1 µM)/ISO (2 µM) or IMD (1 µM, dissolved in normal saline) alone, or in combination. For inhibitor experiments, cells were pre-incubated with a selective autophagy inhibitor 3-MA (10 mM), a PI3K inhibitor wortmannin (100 nM), a PKA inhibitor H89 (10 µM), or a MAPK inhibitor PD98059 (50 µM), respectively, and then treated with Ang II or ISO pre-incubated with either IMD or normal saline together.

### Measurement of Cell Size

H9c2 cardiomyocytes were grown on glass coverslips, and then fixed with 4% formaldehyde solution. After stained with Alexa Fluor 568-conjugated phalloidin (Molecular Probes, Invitrogen, USA) (1∶50 dilution) for 30 min at 37°C to visualize F-actin, at least 50 cardiomyocytes in each group was collected and measured the surface area in three independent experiments by use of Image J program (NIH). The results were expressed as µm^2^ [21].

### Fluoresecence Microscope Imaging of Autophagy after GFP-LC3 Transient Transfection

By use of lipofectamine 2000 (Invitrogen, 11668027), H9c2 cells were transfected with a pEGFP-LC3 plasmid following the manufacturer’s instructions [22]. After treatments, the cells were fixed with 4% paraformaldehyde. The GFP-LC3 punctate dot structures in individual live H9c2 cells were imaged using a fluorescence microscope. Autophagy was quantified by calculating the percentage of GFP-LC3-positive autophagic vacuoles or cells with LC3 punctate dots. Twenty fields of ×600 magnification with 20 to 30 GFP-labeled green cells per field were counted in each condition.

### cAMP Assays

To measure intracellular cAMP level, H9c2 cells were collected after different treatments and lysed in Cell Lysis Buffer (R&D Systems, MN, USA) after multiple freeze-thaw cycles. The suspension was centrifuged (4 min, 3,000×g), and the supernatant was assayed for cAMP content by ELISA (R&D Systems, MN, USA) following the manufacturer’s protocol. Data were normalized to the total protein contents in cell lysates which were measured by Bradford assay (Bio-Rad).

### Reverse Transcription Polymerase Chain Reaction (RT-PCR) and Quantitative Real-Time PCR (qPCR) Assays

Total tissue RNAs of the control, TAC and IMD-treated mice were extracted with TRIzol reagent [17]. For H9c2 cells, total RNA was isolated after 24 h Ang II or ISO stimulations with or without IMD protection, respectively. 1 µg of total mouse or cellular RNA was treated with DNaseI and then primed with a dT oligonucleotide and reverse transcribed with Superscript II. Subsequently, the cDNA was used for RT-PCR amplification by use of a Taq DNA polymerase, and the PCR products were analyzed on a 1% agarose gel stained with ethidium bromide. qPCR reactions were prepared in SYBR Green Supermix for real-time assays. DNA targets were amplified with use of different primers (Table 1) and monitored with a Chromo4 Real-Time PCR Detection System (Bio-Rad Life Sciences). The ANP, BNP, IMD, CRLR, RAMP1, RAMP2 and RAMP3 mRNA levels were normalized to GAPDH. No significant differences in GAPDH levels were observed between different samples when the same amount of total RNA was used (data not shown).

**Table 1:** Sequences of the oligonucleotides used for RT-PCR and qPCR amplification in mouse cardiac tissues and H9c2 cells.

Gene		Primer sequences	Products
ANP	Sense	5′-ATCACCCTGGGCTTCTTC-3′	456 bp
	Antisense	5′-CCTCCTTGGCTGTTATCTT-3′	
BNP	Sense	5′-AAAGTCGGAGGAAATGGC-3′	421 bp
	Antisense	5′-GCTATGTTTATTATGTTGTGGC-3′	
IMD	Sense	5′-CACGACCTGACCCACAAG-3′	169 bp
	Antisense	5′-ATGGCTATGCTGGAATGA-3′	
CRLR	Sense	5′-ATGGCTATGCTGGAATGA-3′	191 bp
	Antisense	5′-TCAGGGCTGTCTTCACTT-3′	
RAMP1	Sense	5′-TGGAGACTATTGGGAAGACG-3′	115 bp
	Antisense	5′-CTGGGATACCTACACGATGC-3′	
RAMP2	Sense	5′-CCTCGCCATCTCACCCAA-3′	195 bp
	Antisense	5′-GGAAGCCCAGCCCAAACT-3′	
RAMP3	Sense	5′-CAACGAGACAGGGATGCT-3′	248 bp
	Antisense	5′-TACACGATGAACTCCGACA-3′	
GAPDH	Sense	5′-TGCCACTCAGAAGACTGTGG-3′	233 bp
	Antisense	5′- GTCCTCAGTGTAGCCAGGA-3′	

### Western Blot Analysis

Western blot assay was performed following the method used as described [22]. The tissues or cells were lyzed at 4°C and then the supernatants were collected after centrifugation. The protein concentration was determined by Bradford assay (Bio-Rad). Protein lysate was loaded on and separated by a 12% SDS-PAGE, and then transferred to polyvinylidene fluoride membrane. The membranes were probed with antibodies against caspase-3, LC3B and β-actin, respectively. Signals were amplified and observed with horseradish peroxidase (HRP)-conjugated secondary antibody (Santa Cruz, CA, USA, 1∶1000 dilution) and enhanced chemiluminescence. An ECL Western Blot Detection System (EMD Millipore, MA, U.S.A.) was used to detect densitometry. All experiments were repeated three times.

### Statistical Analysis

Results were shown as mean±SEM. Statistical analyses between groups of two were done by unpaired t-test. Groups of three or more were analyzed by use of one-way ANOVA, followed by the Newman-Keuls multiple comparison test. A p-value <0.05 was considered statistically significant.

## Results

### IMD and its Receptor Levels in Hypertrophic Cardiac Tissues and H9c2 Cells

**Figure 1:**
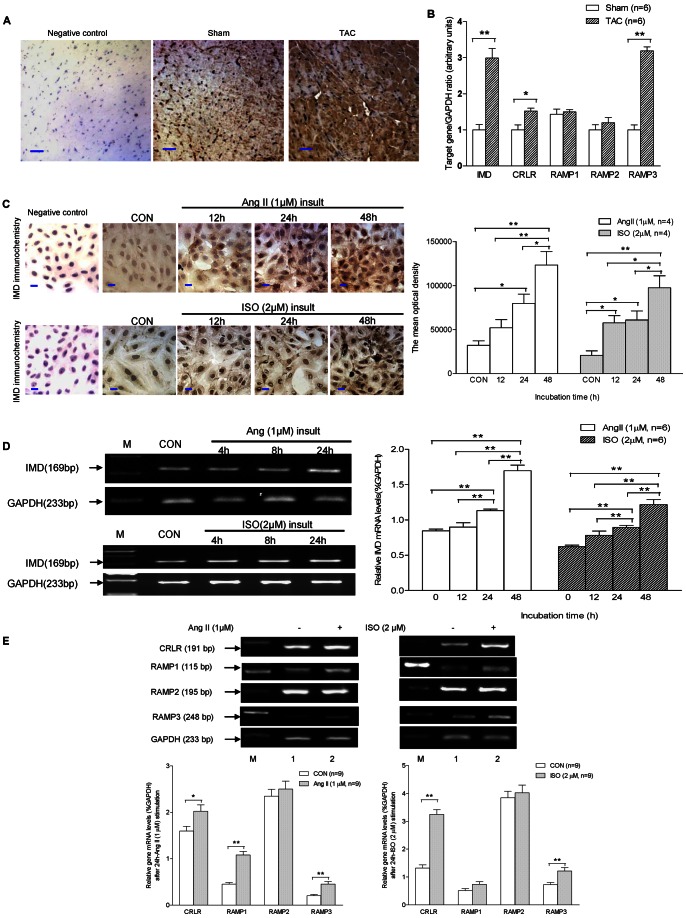
Changes in expression of endogenous IMD and its receptors in TAC-treated cardiac tissues and Ang II or ISO-exposed H9c2 cells. A) The immunostain of IMD protein in mouse cardiac ventricles (n = 4/group). Representative photomicrographs (×400, the scale bar = 50 µm) showed that after TAC, a much stronger positive stain of IMD presented in the cytoplasm of myocardial cells than in that of the sham-surgery control. No any positive stain was observed in negative control without the primary antibody incubation. B) Changes in IMD, CRLR, RAMP1, RAMP2 and RAMP3 gene expression in cardiac tissues induced by TAC (n = 6/group), the data were statistically compared by using the unpaired *t*-test. C) The immunostain of IMD protein in H9c2 cells (n = 4/group). Representative photomicrographs (×400, the scale bar = 50 µm) showed that the positive stain of IMD (measured by the mean optical density) induced by 1 µM Ang II or 2 µM ISO insult was gradually increased in a time-dependent way, the data were analyzed with One-Way ANOVA. In addition, the negative control treated without the primary antibody showed no any positive IMD stain. D) and E) Changes in IMD, CRLR, RAMP1, RAMP2 and RAMP3 gene expression in H9c2 cells stimulated with 1 µM Ang II or 2 µM ISO (n = 6/group). The data (expressed as ratios of target genes and GAPDH gene) are presented as mean ±S.E.M, IMD gene expression data were analyzed with One-Way ANOVA, and the mRNA expression data of CRLR and RAMPs were compared by using the unpaired *t*-test. CON means that mice received sham surgery with vehicle treatment for four weeks, or H9c2 cells without any inducer; TAC, mice received transverse aortic constriction surgery for four weeks. *p<0.05; **P<0.01. IMD, intermedin; M, marker; CRLR, calcitonin gene-related peptide; RAMP, receptor activity-modifying protein.

Cardiac myocytes in control hearts that received sham-surgery (Figure 1A) showed sporadic, positive IMD immunoreactive staining in the cytoplasm. All myocytes in the hearts received TAC (Figure 1A) showed intense, positive IMD staining in the cytoplasm. The mean optical density (96601±27224 vs. 668741±184296, both n = 4) was 6.9-fold (P<0.01) higher, than those in the control group. We further detected the mRNA expression of IMD, CRLR, RAMP1, RAMP2 and RAMP3 gene in cardiac tissues by use of qPCR assay, and observed that IMD, CRLR and RAMP3 mRNA levels were significantly much higher in TAC-treated mice than the controls, by 3.0-fold (P<0.01), 1.5-fold (P<0.05) and 3.2-fold (P<0.01), respectively (Figures 1B). However, there were no significant differences in the mRNA expression levels of RAMP1 and RAMP2 between the mice that received TAC and sham surgery (both P>0.05, Figures 1B).

In H9c2 cells, the IMD immunostain was sporadically distributing in cytoplasm of control cells without any inducer, while gradually augmented in a time-dependent manner with Ang II (1 µM) or ISO (2 µM) insult, with increase in mean optical density by 0.6 (P>0.05), 1.5 (P<0.05) and 2.9-fold (P<0.01), or 2. 8 (P<0.05), 2.9 (P<0.05) and 4.7-fold (P<0.01), 12, 24 and 48 h after the stimulation, respectively, compared with the controls (Figure 1C). In addition, the IMD mRNA level showed a time-dependent increase in H9c2 cells with Ang II or ISO exposure (Figure 1D), with 6.4% (P>0.05), 34.5% (P<0.01) and 100.7% (P<0.01), or 25.9%(P>0.05), 44.0% (P<0.01) and 94.9% (P<0.01) higher, 12, 24 and 48 h after the stimulation, respectively, than the controls. Furthermore, the mRNA expression of CRLR, RAMP1 and RAMP3 was significantly increased in 24 h Ang II-insulted cells (Figure 1E) by 27.0% (P<0.05), 2.4-(P<0.01) and 2.2-fold (P<0.01), respectively, compared with the controls. CRLR and RAMP3 gene expression in 24 h ISO-treated cells was 2.5 (P<0.01) and 1.7-fold (P<0.01) higher than the controls, respectively. While, no obvious change was observed in RAMP2 mRNA levels between the controls and the Ang II or ISO insulted cells. In addition, no any active IMD staining was observed in any tissue or cell sections of negative controls treated with non-special rabbit IgG, normal goat serum instead of primary antibody (Figures 1A, C).

### IMD Improved the Left Ventricular Dysfunction Induced by TAC

**Figure 2:**
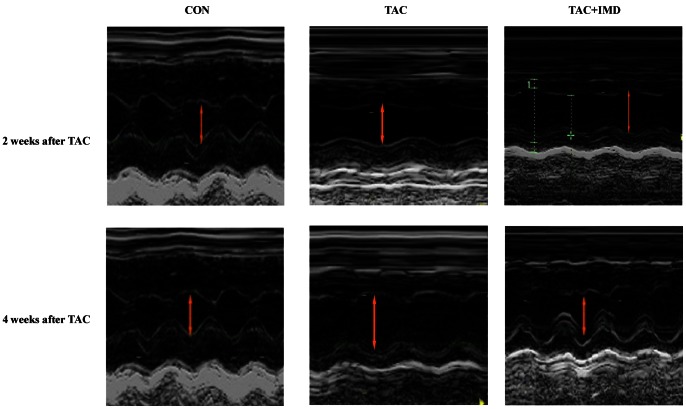
Effect of TAC and IMD supplementation on left ventricular wall and chamber dimensions. Representative M-mode frames from the mid-papillary region of CON, TAC and TAC+IMD 2 and 4 weeks after the beginning of TAC treatment. Yellow arrow indicates width of the LV chamber. CON means that mice received sham surgery with vehicle treatment for two or four weeks; TAC, mice received transverse aortic constriction surgery for two or four weeks; TAC+IMD, TAC mice received subcutaneous IMD (200 ng/kg/hour) treatment with a mini-osmotic pump for two or four weeks. IMD, intermedin.

To evaluate the cardiac protection of IMD, echocardiography was performed 2 and 4 weeks after the TAC surgery (Figure 2 and Table 2). 2-week after TAC, we observed that compared with the control, TAC treated animals showed a tendency of increase in left ventricle (LV) posterior wall dimension (LVPWd) and stroke volume (SV), and a reduction tendency in left ventricle end systolic diameter (LVSd), LV internal dimension in diastole (LVIDd), ejection fraction (EF) and left ventricular fractional shortening (FS) (all P>0.05), respectively, which indicated a compensatory concentric hypertrophy. However, there was no statistical difference in the echocardiographic parameters among the control, TAC surgery or IMD supplementation animals. 4 weeks after TAC, the cardiac dysfunction was deteriorated, with increase in LVSd, LVIDd, LVPWd, end-diastolic volume (EDV) and end-systolic volume (ESV) by 28.6%, 25.9%, 26.9%, 40.0% and 166.7%, and decrease in EF, FS and SV by 16.8%, 18.9% and 33.3%, respectively, compared with the control (all P<0.01); these parameters were also 30.4% (P<0.01), 25.9% (P<0.01), 26.9% (P<0.05), 40.0% (P<0.01) and 33.3% (P<0.01) more, and 13.2% (P<0.01), 8.9%, 42.9% (P<0.01) less, than TAC 2 weeks group. These results indicated congestive heart failure with left ventricular dilatation occurring at 4 weeks of TAC treatment. IMD treatment remarkably improved the cardiac functions, with LVSd, LVIDd, LVPWd, EDV and ESV decreased by 16.7% (P<0.01),16.8% (P<0.01),12.9% (P<0.05),14.3% (P<0.01) and 50.0% (P<0.01), and EF, FS and SV increased by 10.6% (P<0.05), 18.7% (P<0.01) and 25.0% (P>0.05), compared to TAC 4 weeks group.

**Table 2:** Two-Dimensional Echocardiographic Analysis of Left Ventricular Wall and Chamber Dimension at 2 weeks or 4 weeks after TAC in Control, TAC alone and together with IMD supplementation treated Mice (mean±s.e.m., n = 15).

	CON	2-week after TAC		4-week after TAC	
		TAC	TAC+IMD	TAC	TAC+IMD
LVSd(mm)	0.70±0.02	0.69±0.02	0.73±0.01	0.90±0.04**##	0.79±0.03* &&
LVIDd(mm)	3.40±0.11	3.37±0.10	3.42±0.06	4.28±0.13**##	3.56±0.10&&
LVPWd(mm)	0.67±0.02	0.72±0.05	0.71±0.005	0.85±0.04**#	0.74±0.02&
LVIDs(mm)	2.25±0.07	2.25±0.03	2.30±0.08	2.51±0.11	2.28±0.09
EDV(ml)	0.10±0.003	0.10±0.005	0.10±0.003	0.14±0.008**##	0.12±0.003* &&
ESV(ml)	0.03±0.003	0.03±0.005	0.03±0.005	0.08±0.008**##	0.04±0.001&&
EF(%)	70.4±1.78	67.5±1.96	67.3±1.47	58.6±1.83**##	64.8±2.05* &
%FS	34.3±1.13	30.5±0.98	32.7±1.06	27.8±0.85**	33.0±1.16&&
SV(ml)	0.06±0.003	0.07±0.005	0.06±0.003	0.04±0.003**##	0.05±0.005

LVSd, left ventricle (LV) end systolic diameter; LVIDd, LV internal dimension in diastole; LVPWd, LV posterior wall dimension; LVIDs, LV internal dimension in systole; EDV, End-diastolic volume; ESV, end-systolic volume; EF(Teich)%, ejection fraction calculated by using the Teichholz method; FS%, Left ventricular fractional shortening %; SV(Teich), stroke volume calculated by using the Teichholz method. CON, control; TAC and TAC+IMD mean mice received transverse aortic constriction treatment without and with IMD treatment, respectively.

* P<0.05 and **P<0.01 vs the control;

# P<0.05,

## P<0.01 vs TAC alone 2 weeks group;

& P<0.05,

&& p<0.01 vs TAC alone 4 weeks group. TAC, transverse aortic constriction; IMD, intermedin.

We further detected the effect of IMD on cardiac function by use of a microtip pressure catheter (Table 3). Compared with the control animals, TAC-treated 4 weeks mice presented a significant change in haemodynamic parameters, with left ventricle pressure (LVP), LV end-systolic pressure (LVESP), the maximal value of the first derivative of LV pressure (dP/dt_max_), the minimal value of dP/dt (dP/dt_min_), SV, cardiac output (CO), EF, stroke work (SW) and heart rate decreased by 13.9% (P<0.05), 11.6% (P<0.05),11.9% (P<0.01), 9.7% (P>0.05), 27.5% (P<0.01), 30.3% (P>0.05), 18.8% (P<0.01), 32.1% (P<0.05) and 14.2% (P<0.05), while LV end-diastolic pressure (LVEDP), ESV, EDV and earterial elastance (AE) increased by 227.9% (P<0.01), 37.7% (P<0.01), 11.1% (P>0.05) and 35.2% (P<0.01), respectively. IMD supplementation significantly improved the cardiac performance, with LVP, LVESP, dP/dt_max_, dP/dt_min_, SV, CO, EF, SW and heart rate increased by 8.6% (P>0.05), 7.8% (P>0.05), 10.6% (P<0.05), 11.1% (P<0.05), 28.2% (P<0.05), 26.8% (P<0.05),15.0% (P>0.05), 29.8% (P<0.05) and 12.4% (P<0.05), but LVEDP, ESV, EDV and AE decreased by 49.3% (P<0.01), 25.4% (P<0.01), 7.6% (P>0.05) and 22.8% (P<0.01), respectively, compared to TAC alone group.

**Table 3:** Left Ventricular Function in Control, TAC alone and together with IMD supplementation treated Mice (mean±s.e.m., n = 10).

	CON	TAC	TAC+IMD
LVP(mmHg)	91.6±3.3	78.9±3.0*	85.7±3.6
LVESP(mmHg)	93.80±3.1	82.90±3.3*	89.40±2.9
LVEDP(mmHg)	2.1±0.1	6.8±0.2**	3.5±0.1**##
dP/dt_max_(mmHg/s)	6,154±160	5,419±170**	5,992±144#
dP/dt_min_(mmHg/s)	−5,270±154	−4,757±134	−5,287±190
ESV(µl)	29.2±1.3	40.2±1.8**	30.0±1.2##
EDV(µl)	46.1±1.6	51.2±2.1	47.3±1.5
SV(µl)	17.1±1.1	12.4±0.9*	15.9±1.3#
CO(µl/min)	7,859±463	5,476±340**	6,945±475#
EF(%)	63.3±2.4	51.4±2.0**	59.1±2.6#
SW(mmHg×µl)	1,882±172	1,277±145*	1,658±156
AE(mmHg/µl)	9.2±0.3	12.4±0.3**	9.6±0.4##
Heart rate(bpm)	479±16	411±12*	462±19#

LVP, left ventricle (LV) pressure; LVESP, LV end-systolic pressure; LVEDP, LV end-diastolic pressure; dP/dt_max_, maximal value of the first derivative of LV pressure; dP/dt_min_, minimal value of the first derivative of LV pressure; ESV, end-systolic volume; EDV, End-diastolic volume; SV, stroke volume; CO, cardiac output; EF, ejection fraction; AE, arterial elastance; SW, stroke work. CON, control; TAC and TAC+IMD mean mice received transverse aortic constriction treatment without and with IMD treatment for 4 weeks, respectively.

* P<0.05 and **P<0.01 vs the control;

# P<0.05,

## P<0.01 vs TAC alone 4 weeks group. TAC, transverse aortic constriction; IMD, intermedin.

### IMD Attenuated Cardiac Hypertrophy and Remodeling Induced by TAC

**Figure 3:**
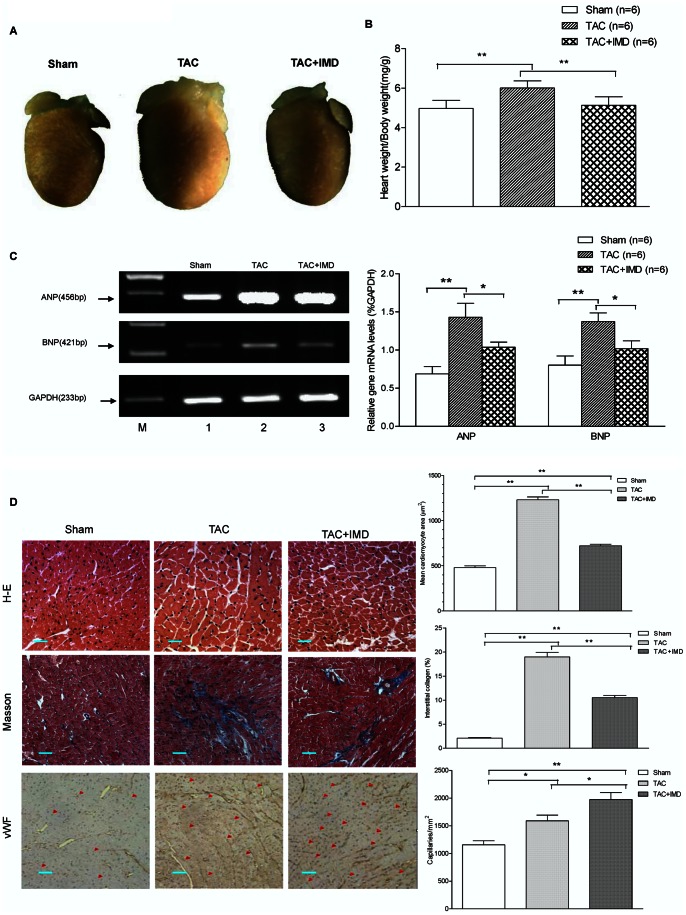
Protection of IMD supplementation against TAC-induced cardiomyocyte hypertrophy, interstitial fibrosis, and capillary angiogenesis. A) Representative pictures of hearts from sham-operated control and TAC mice treated with vehicle or IMD. B) Heart weight/body weight ratio of sham-operated control and TAC mice treated with vehicle or IMD (n = 6). C) Changes in mRNA level of ANP and BNP in cardiac tissues of sham-operated control and TAC mice treated with vehicle or IMD (n = 6). The data (expressed as ratios of target genes and GAPDH gene) are presented as mean ±S.E.M. D) Representative H&E (×400, scale bars = 50 µm), Masson trichrome (×400, scale bars = 50 µm) and vWF immunohistology (×200, scale bars = 100 µm) stained photomicrographs exhibiting cross-sectional cardiomyocyte area, myocardial fibrosis and capillary, respectively. Quantitative analysis of cardiomyocyte cross-sectional area with measurements of 100–150 cardiomyocytes from 4 mice per group, fibrotic area with normalizing blue Masson trichrome–stained area to total myocardial area from 30–50 randomly chosen frames from 4 mice per group, capillary density with counting vWF antibody-stained microvessels from 10 random fields per section (n = 4/group). The negative control for vWF immunohistology stain was the same as that for IMD stain in Figure 1A, there was no any positive vWF stain. CON means that mice received sham surgery with vehicle treatment for four weeks; TAC, mice received transverse aortic constriction surgery for four weeks; TAC+IMD, TAC mice received subcutaneous IMD (200 ng/kg/hour) treatment with a mini-osmotic pump for four weeks. All data were statistically analyzed with One-Way ANOVA. *p<0.05; **P<0.01. IMD, intermedin; ANP, atrial natriuretic peptide; BNP, brain natriuretic peptide.

Cardiac hypertrophy is histologically characterized by increased myocyte mass (hypertrophy) and extracellular matrix deposition (fibrosis), as well as capillary and pre-capillary coronary vascular growth [1], [2]. By gross morphologic examination, it was observed that TAC induced a significant increase in heart weight/body weight ratios (HW/BW) and the mRNA levels of A-type natriuretic peptide (ANP) and brain natriuretic peptide (BNP), the important cardiac hypertrophic markers, with 20.96%, 107.9% and 71.6% higher, respectively, than the control (all P<0.01, Figure 3A, B and C). IMD-supplementation significantly reduced the TAC-increased HW/BW, ANP and BNP mRNA levels, with 17.0% (P<0.01), 37.9% (P<0.05) and 34.7% (P<0.05) less, respectively, than TAC alone group (Figure 3A, B and C). In addition, in hematoxylin and eosin (HE) staining of the myocardium, the myocyte cross-sectional area was significantly increased after TAC treatment by 156.3%, compared with the control, while decreased in IMD-treated TAC mice by 70.8%, compared to TAC alone mice (both P<0.01, Figure 3D). In Masson staining sections, TAC-treated mice was accompanied by extensive interstitial fibrosis in the ventricular wall by 8.1-fold more than the control hearts (P<0.01, Figure 3D). IMD supplementation remarkably attenuated ventricular fibrosis by 81.0%, compared with TAC alone group (P<0.01, Figure 3D). These results indicated that the IMD showed a potent antagonization of cardiac hypertrophy and remodeling induced by pressure overloading.

Myocardial angiogenesis with pressure-overload hypertrophy is important in maintaining maximal left ventricle coronary flow [23]. We evaluated capillary density in heart by performing immunohistochemistry for vWF in cardiac tissue sections. Compared with the control, capillary density was significantly increased by 37.4% in TAC-treated mouse hearts (P<0.05), which was augmented by IMD supplementation, with 24.1% higher than TAC alone group (P<0.05). The results indicated that IMD induced adaptive capillary growth in left ventricular pressure overload hypertrophy to improve myocardial blood supply.

**Figure 4:**
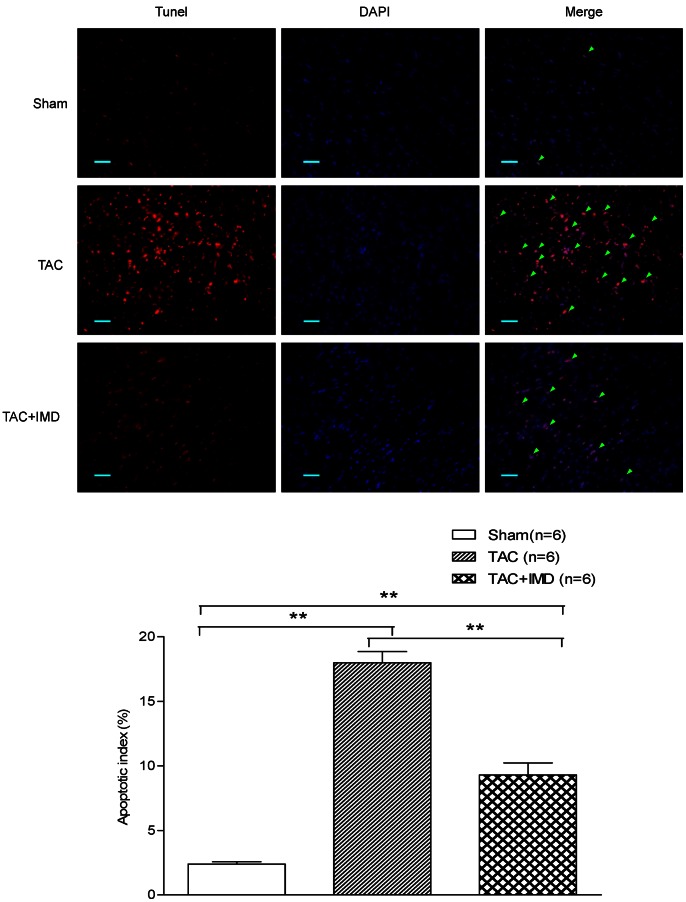
Effect of IMD supplementation on myocardial apoptosis after TAC treatment. Immunofluorescent TUNEL stained heart tissue sections from sham-operated control and TAC mice treated with vehicle or IMD (n = 6/group). Green arrows indicate some purplish red-stained TUNEL-positive nuclei. Scale bar = 50 µm. Original magnifications, ×400. CON means that mice received sham surgery with vehicle treatment for four weeks; TAC, mice received transverse aortic constriction surgery for four weeks; TAC+IMD, TAC mice received subcutaneous IMD (200 ng/kg/hour) treatment with a mini-osmotic pump for four weeks. The data were statistically analyzed with One-Way ANOVA. *p<0.05; **P<0.01. IMD, intermedin.

Cardiac hypertrophy is associating with cardiac cell loss induced by apoptosis [2], which contributes to cardiac dysfunction. We observed that TUNEL-positive nuclei significantly increased 4 weeks after TAC by 7.5–fold, comparing with the control (18.0±2.1% vs 2.4±0.4%, p<0.01). IMD supplementation (9.3±2.3%) remarkably decreased the apoptotic index, with 3.9-fold (p<0.01) less than TAC alone group (Figure 4).

### IMD Attenuated Hypertrophy in H9c2 Cells

**Figure 5:**
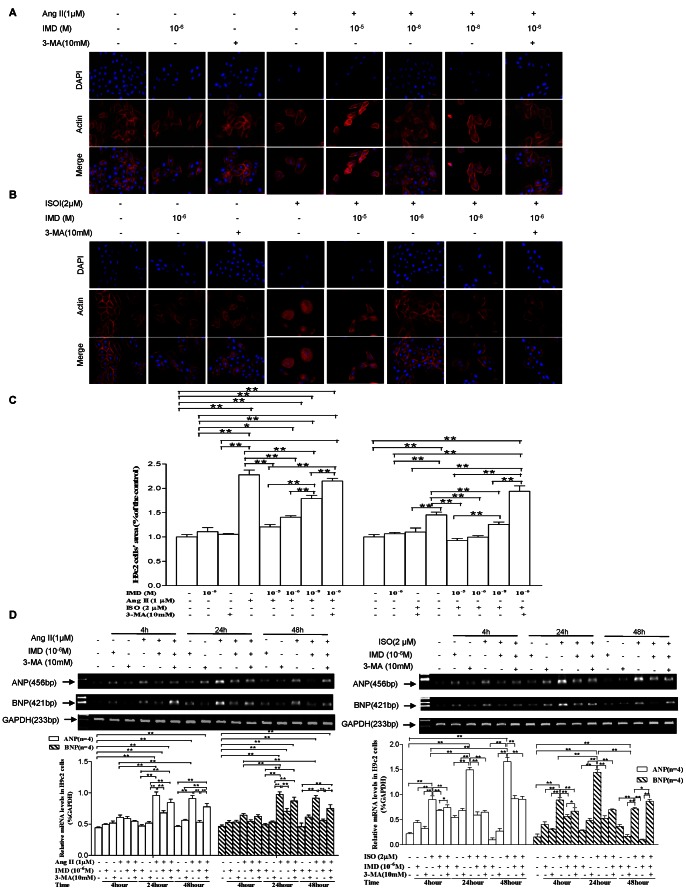
IMD supplementation inhibited Ang II or ISO-induced H9c2 cell hypertrophy. A) and B) Representative red F-actin stained photomicrographs. H9c2 cells were incubated with 1 µM Ang II or 2 µM ISO in the absence or presence of 10^−8^–10^−5^M IMD, or 10 mM 3-MA alone or together with both 10^−6^ M IMD and Ang II or ISO, respectively, for 48 h, and then cell area was assayed with Alexa Fluor 568-conjugated phalloidin staining. Scale bars = 50 µm. Original magnifications, ×400. C) Quantitative analysis of H9c2 cell size with measurements of ≥50 cardiomyocytes per group. D) Changes in mRNA level of ANP and BNP in H9c2 cells treated with 10^−6^ M IMD, 1 µM Ang II or 2 µM ISO alone or in combination, or 10 mM 3-MA alone or together with both 10^−6^ M IMD and Ang II or ISO, for 4, 24 and 48 h, respectively (n = 4/group). The data (expressed as ratios of target genes and GAPDH gene) are presented as mean ±S.E.M. All data were statistically analyzed with One-Way ANOVA. *p<0.05; **P<0.01. IMD, intermedin; Ang II, angiotensin II; ISO, isoproterenol; 3-MA, 3-methyladenine; ANP, atrial natriuretic peptide; BNP, brain natriuretic peptide.

To verify the effect of IMD on cardiac hypertrophy at cellular level, H9c2 cells were treated with either Ang II (1 µM) or ISO (2 µM), with or without addition of IMD supplementation (Figure 5A, B and C). Both Ang II and ISO exposure for 48 h induced significant increase in myocyte size, with 127.7% and 45.3% (both P<0.01) more, respectively, than the controls. Pre-incubation of IMD for 30 min and then co-incubation with Ang II or ISO for another 48 h remarkably reduced the cell size in a concentration dependent manner, with 47.1% (P<0.01), 38.3% (P<0.01) and 21.1% (P>0.05), or 36.1% (P<0.01), 31.5% (P<0.01) and 13.7% (P<0.01) less in IMD 10^−5^, 10^−6^ and 10^−8 ^M co-incubation groups, respectively, than Ang II or ISO alone group (Figure 5C). Interestingly, pre-incubation with 3-MA, an autophagy inhibitor, for 30 min and then co-incubation with both IMD and Ang II or ISO for another 48 h, the cell size was significantly increased by 52.8% or 94.6%, respectively, compared with IMD together with Ang II or ISO group (both P<0.01). In addition, IMD or 3-MA incubation alone showed no obvious effect on cell size (both P>0.05). Hypertrophic markers, mRNA expression of ANP and BNP, were further detected. Compared with the controls, both ANP and BNP expression were increased significantly 24 and 48 h after Ang II exposure, respectively, which was remarkably reduced by IMD pre-incubation for 30 min and then co-incubation with Ang II for another 24 and 48 h, compared to Ang II alone groups. While, 3-MA pre-incubation for 30 min and then co-incubation with both IMD and Ang II, increased both ANP and BNP mRNA level at 48 h by 46.3% and 35.6% (both P<0.01), respectively, compared with IMD together with Ang II group (Figure 5D). Similar results were observed in ISO insulted H9c2 cells. Both ANP and BNP expression were markedly induced by ISO exposure for 4, 24 and 48 h, respectively, compared with the controls. Both of them were significantly decreased by IMD pre-incubation for 30 min and then co-incubation with ISO for another 4, 24 and 48 h, compared to ISO alone groups. 3-MA pre-incubation for 30 min and then co-incubation with both IMD and ISO, augmented their mRNA expression by 8.2% and 18.8% at 4 h (both P<0.5), and BNP mRNA level by 7.8-fold at 48 h (P<0.01), respectively, compared with IMD together with ISO groups (Figure 5D). In addition, IMD or 3-MA incubation alone for 4, 24 and 48 h showed no obvious effect on both ANP and BNP mRNA expression (all P>0.05, Figure 5D).

### IMD Protected Hypertrophy-induced Cardiomyocyte from Apoptosis through Activation of Autophagy

**Figure 6:**
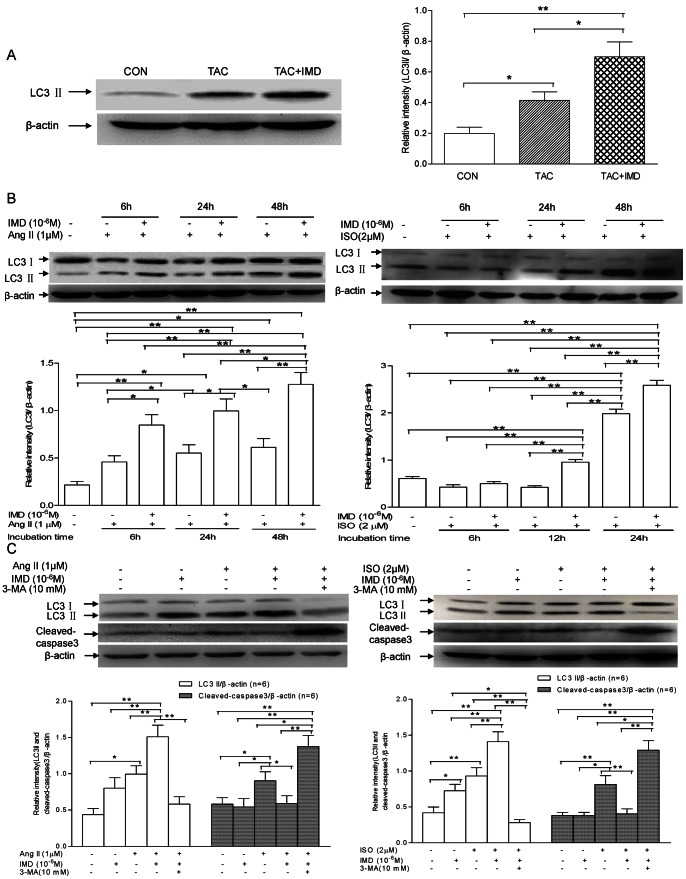
Effect of IMD supplementation on autophagy level in mouse heart tissues and H9c2 cells. A) LC3I, LC3II and β-actin levels in mouse hearts (n = 6/group) treated with TAC alone or in combination with exogenous IMD, respectively. Densitometric analysis showed that IMD supplementation significantly augmented the TAC-induced LC3II/β-actin ratios. B) LC3I, LC3II and β-actin levels in H9c2 cells (n = 6/group) incubated with 1 µM Ang II or 2 µM ISO in the absence or presence of 10^−6^M IMD for 6–48 h, respectively. Densitometric analysis showed that IMD supplementation significantly augmented the LC3II/β-actin ratios in cells exposed to Ang II or ISO. C) LC3I, LC3II, capase3 and β-actin levels in H9c2 cells (n = 6/group) incubated with either Ang II (1 µM)/ISO(2 µM) or in combination with 10^−6^M IMD in the absence or presence of 10 mM 3-MA for 24 h, respectively. Densitometric analysis showed that IMD supplementation remarkably augmented Ang II or ISO-induced LC3II/β-actin ratios and suppressed the stirred cleaved capapse3/β-actin ratios, and 3-MA almost abrogated the protective effects of IMD against apoptosis by suppressing the autophagy induced by IMD. CON means that mice received sham surgery with vehicle treatment for four weeks; TAC, mice received transverse aortic constriction surgery for four weeks; TAC+IMD, TAC mice received subcutaneous IMD (200 ng/kg/hour) treatment with a mini-osmotic pump for four weeks. All data were statistically analyzed with One-Way ANOVA. *p<0.05; **P<0.01. IMD, intermedin; Ang II, angiotensin II; ISO, isoproterenol; 3-MA, 3-methyladenine.

Autophagy is involved in the most important cardiac pathologies including myocardial hypertrophy. The regulation of survival and death by autophagy and apoptosis is rather complex [6]. The activation kinetics of autophagy and apoptosis were examined to reveal potential signaling pathways involved in the antiapoptotic function of IMD. LC3-II, localized in both the outer and inner membranes of the autophagosome, is usually utilized as a special autophagosome marker [11]. Tracking the conversion of LC3-I to LC3-II is indicative of autophagic activity [12]. As shown in Figure 6 A, TAC induced much higher LC3-II level in mouse hearts, with the ratios of LC3-II to β-actin increased by 109.4%(P<0.05), compared with the control animals. In H9c2 cells, 1 µM Ang II alone treatment for 6, 24 and 48 h induced gradually higher level of LC3-II, with 112.7% (P<0.05), 156.4% (P<0.05) and 2.1-fold (P<0.01) more LC3-II/β-actin ratio than the control, respectively (Figure 6B). While 2 µM ISO exposure decreased the level of LC3-II at 6 and 12 h, and significantly increased it at 24 h, with 30.49% (P>0.05) and 31.1% (P>0.05) less, and 2.2-fold (P<0.01) higher in LC3-II/β-actin ratio, than the control, respectively (Figure 6B). IMD treatement markedly increased LC3-II level, with 68.4% higher in cardiac tissues (P<0.05, Figure 6A), 84.6%(P>0.05), 80.4% (P<0.05), 107.9% (P<0.01) higher at 6, 24 and 48 h in Ang II-insulted cells, and 17.7%(P>0.05), 127.4%(P<0.01), 30.6%(P<0.01) higher at 6, 12 and 24 h in ISO-treated cells, respectively, than TAC, Ang II or ISO alone groups (Figure 6B).

To reveal the functional relationship between autophagy and apoptosis, we further detected active caspase-3 level. As shown in Figure 6C, in H9c2 cells, Ang II and ISO insults for 24 h induced much higher LC3-II and cleaved capase-3 levels, with increase in LC3-II/β-actin ratios by 128.2% (P<0.05) and 122.7% (P<0.01), and cleaved caspase-3/β-actin ratios by 55.4% (P<0.05) and 113.7% (P<0.01), respectively, compared with the controls. IMD pre-incubation for 30 min and then co-incubation with Ang II or ISO for another 24 h, significantly increased the LC3-II/β-actin ratios by 51.7% and 51.4% (both P<0.01), while decreased in caspase-3/β-actin ratios by 34.6% (P<0.05) and 50.6% (P<0.01), respectively, compared to Ang II or ISO alone group. Interestingly, when 3-MA was used to block the IMD-augmented autophagy, the significant increase in cardiomyocyte apoptosis was observed. Compared with the cells treated with either Ang II or ISO in-combination with IMD, 3-MA pre-incubation for 30 min and then co-incubation with both IMD and Ang II or ISO for another 24 h, decreased LC3-II/β-actin ratios by 1.6 and 4.0-fold(both P<0.01), while increased capase-3/β-actin ratios by 132.41% and 2.25-fold (both P<0.01), respectively. These results indicated that IMD induced a protective autopahgy against hypertrophic stimuli, to help cardiomyocyte survival.

**Figure 7:**
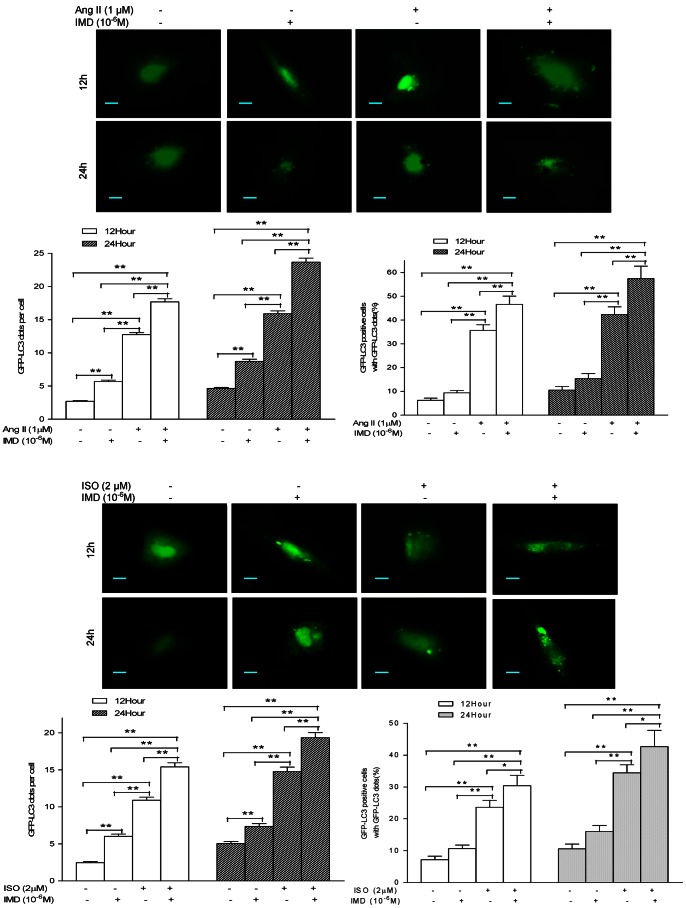
Representative fluorescence images showing the effect of exogenous IMD (10^−6^M) on the appearance of a punctate GFP-LC3 signal stirred by 1 µM Ang II or 2 µM ISO insult in H9c2 cells for 12 and 24 h transfected with a pEGFP-LC3 plasmid, respectively. Positive signals were defined as those cells that had five or more GFP-LC3 dots in the cytoplasm. The percentage of the cells with GFP-LC3 dots and the average number of GFP-LC3 dots per cell were analyzed from at least 100 random fields, and both of them were markedly increased by IMD supplementation at 12 h and 24 h, respectively. All data were statistically analyzed with One-Way ANOVA. *p<0.05; **P<0.01. IMD, intermedin; Ang II, angiotensin II; ISO, isoproterenol.

GFP-LC3 is a marker for autophagic membranes, which allows the direct visualization of autophagy in cells. We transiently transfected a pEGFP-LC3 plasmid into H9c2 cells, as showed in Figure 7, the control cells presented diffused and weak LC3 punctate dots in cytoplasm, while IMD alone slightly induced bright green LC3 punctate dots in the cytoplasm, with increase in average number of GFP-LC3 dots per cell by 111.5% and 87.7% (both P<0.01) at 12 and 24 h in Ang II insult experiment, and 145.7% and 45.8% (both P<0.01) at 12 and 24 h in ISO insult experiment, respectively, compared with the controls (Figure 7). Ang II alone incubation induced a cornucopia of bright green LC3 punctate dots in the cytoplasm, both the average number of GFP-LC3 dots per cell and percentage of cells with GFP-LC3 dots were increased by 3.8-, 2.4-folds and 4.7-, 3.0-fold after 12 and 24 h stimulation, respectively, compared with the controls, while they were further increased by IMD co-incubation for 12 and 24 h, with 38.7%, 48.9% and 30.8%, 35.5% more than those of Ang II-alone groups, respectively (Figure 7, all P<0.01). ISO alone stimulation also induced lots of bright green LC3 punctate dots in the cytoplasm, both the average number of GFP-LC3 dots per cell and percentage of cells with GFP-LC3 dots were increased by 5.3-, 1.9-folds and 3.3-, 3.3-fold after 12 and 24 h treatment, respectively (all P<0.01), compared with the controls. They were also augmented by IMD co-incubation for 12 and 24 h, with 41.3% (P<0.01), 31.0% (P<0.01) and 28.6% (P<0.05), 23.9% (P<0.05) more than those of ISO-alone groups, respectively (Figure 7).

### IMD Stimulated Intracellular cAMP Production in H9c2 Cells

**Figure 8:**
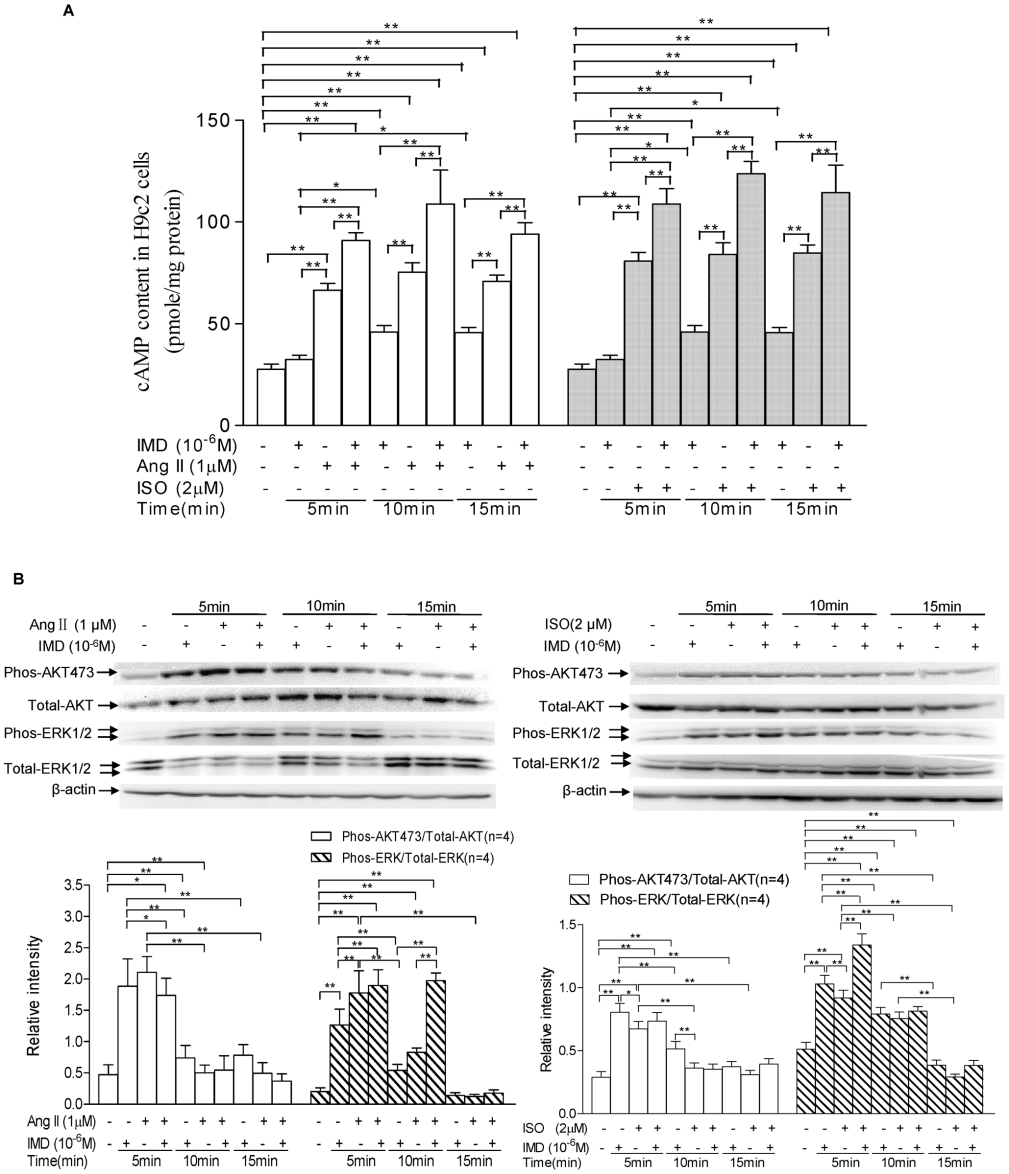
IMD supplementation augments Ang II or ISO-induced intracellular cAMP contents and ERK1/2 phosphorylation in H9c2 cells. A) Effects of IMD on basal and Ang II or ISO-stimulated cAMP production in H9c2 cells. H9c2 cells was exposed to IMD (10^−6^M) alone for 10 and 15 min, or Ang II (1 µM) or ISO (2 µM) alone for 5, 10 and 15 min, respectively, induced a rise of intracellular cAMP formation. IMD co-incubation with Ang II or ISO further augmented the intracellular cAMP contents. B) Regulation of IMD on its receptor-post signaling pathways including Akt and ERK1/2, detected by western blot analysis (n = 4/group). H9c2 cultures were treated with Ang II (1 µM) or ISO (2 µM) in the absence and presence of 10^−6^M IMD for 5, 10 and 15 min, respectively, and then cell lysates were collected. Immunoblots were prepared and probed for phosphorylated (Phos-) and total Akt and ERK1/2. Densitometric analysis showed that compared with Ang II or IMD alone group, IMD supplementation significantly increased the ratio of phosphorylated/total ERK1/2 but showed no obvious effect on the ratio of phosphorylated/total Akt. All data were statistically analyzed with One-Way ANOVA. *p<0.05; **P<0.01. IMD, intermedin; Ang II, angiotensin II; ISO, isoproterenol.

IMD (10^−6^M) alone exposure for 10 and 15 min induced a rise of intracellular cAMP formation in H9c2 cells, with 65.5%, 64.6%, respectively, compared with the controls (all P<0.01, Figure 8A). Ang II (1 µM) or ISO (2 µM) alone stimulation stirred the cAMP production in cells, with 140.0%, 171.7% and 155.4%, or 191.4%, 203.2% and 206.0% higher in cAMP contents at 5, 10 and 15 min of Ang II or ISO exposure, respectively, than the controls (all P<0.01, Figure 8A). IMD co-incubation with Ang II or ISO further augmented the cAMP contents in H9c2 cells, with 36.7%, 44.6% and 32.9%, or 34.8%, 47.3% and 35.1% more cAMP level, 5, 10 and 15 min after both IMD and Ang II or ISO co-incubation, compared with Ang II or ISO alone group (all P<0.01, Figure 8A).

### IMD Activated Autophagy via Activating PKA and MAPK/ERK1/2 Signaling Pathways

**Figure 9:**
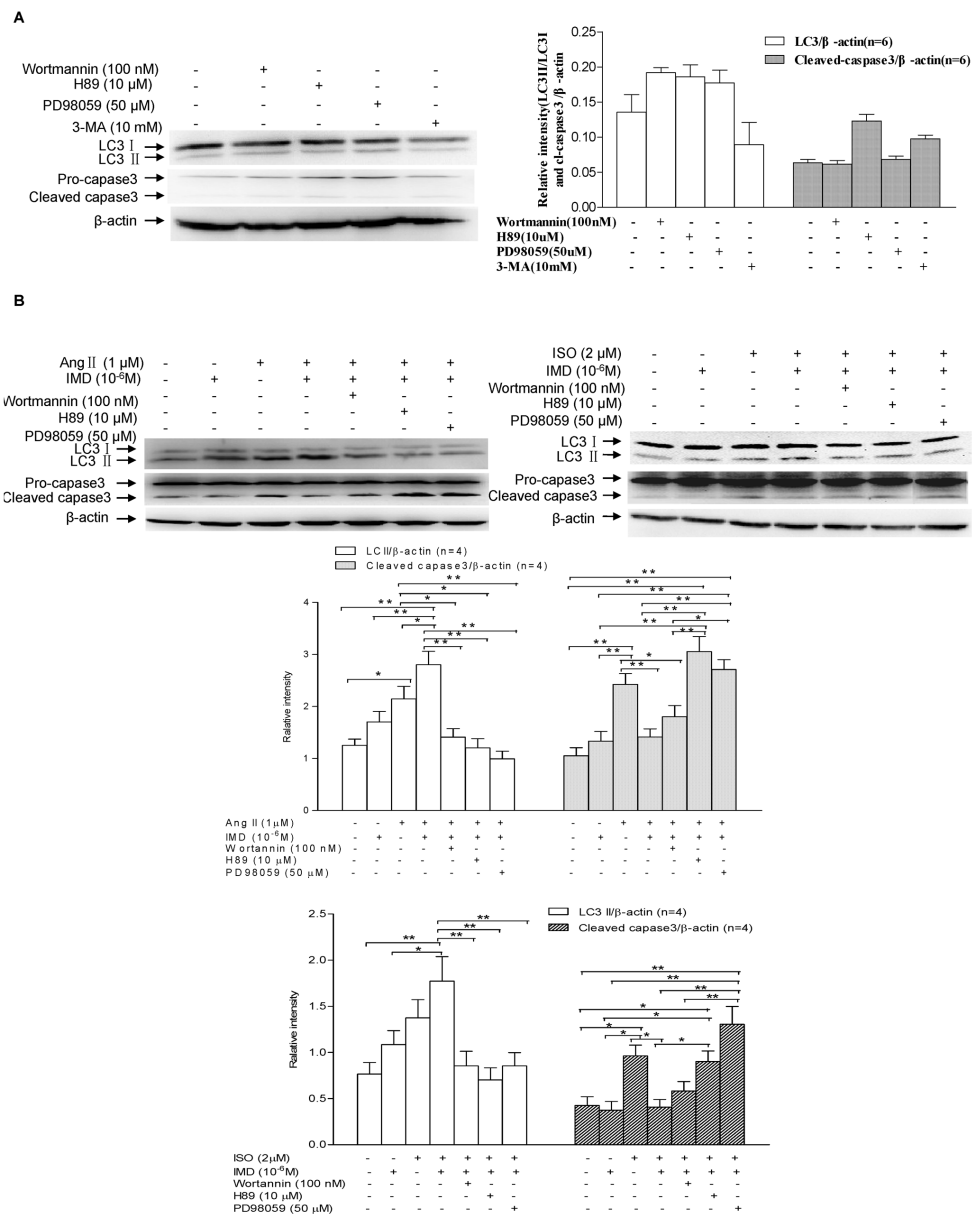
IMD supplementation augments autophagy level induced by Ang II or ISO exposure, and suppresses apoptosis stirred by Ang II or ISO insult in H9c2 cells, involving PKA and MAPK/ERK1/2 activation. A) H9c2 cells were incubated with 100 nM wortmannin (a PI3K inhibitor), 10 µM H89 (a PKA inhibitor), 50 µM PD98059 (a MAPK inhibitor) or 10 mM 3–MA (an autophagy inhibitor) alone for 24 h, respectively, and then LC3I, LC3II, capase3 and β-actin proteins were detected by western blot assay (n = 4/group). Densitometric analysis showed that the inhibitors alone had no obvious effect on the ratios of LC3-II/β-actin and cleaved caspase-3/β-actin, respectively. B) LC3I, LC3II, capase3 and β-actin levels in H9c2 cells (n = 4/group) treated with IMD (10^−6^M) or Ang II (1 µM)/ISO(2 µM) alone, or in combination in the absence and presence of wortmannin(100 nM), H89(10 µM), or PD98059(50 µM) for 24 h, respectively. Densitometric analysis showed that wortmannin, H89 and PD98059 pre-incubation remarkably reduced co-incubation of IMD with Ang II or ISO-induced LC3II/β-actin ratios, while only H89 and PD98059 pre-incubation significantly augmented the cleaved capapse3/β-actin ratios in cells co-incubated with both IMD and Ang II or ISO. All data were statistically analyzed with One-Way ANOVA. *p<0.05; **P<0.01. IMD, intermedin; Ang II, angiotensin II; ISO, isoproterenol; 3-MA, 3-methyladenine.

Three major signaling pathways, PKA, PI3K, and MAPK/ERK1/2, have been identified to transduce the cellular actions of IMD. We have proved that IMD stimulated intracellular cAMP production, which can activate cAMP-Dependent PKA. Here, we further investigated the role of PI3K and MAPK/ERK1/2 pathways in the anti-apoptotic effects of IMD. As showing in Figure 8B, IMD incubation alone significantly induced a rapid activation (5 min) in AKT and ERK1/2 kinases, a sustained increase in their phosphorylation lasting at least 10 min, with phospho-Akt (Serine 473)/total Akt ratios higher by 3.0-fold (P<0.01), 55.3% (P<0.01), 66.0% (P<0.05) in Ang II insult experiment, and 175.9% (P<0.01), 75.9% (P<0.01), 27.6% (P>0.05) in ISO insult experiment, after Ang II or ISO exposure for 5, 10 and 15 min, respectively, than the controls; in addition, phospho-ERK1/2/total ERK1/2 ratios was also observed much higher by 5.3-fold (P<0.01), 170.0% (P<0.01), in Ang II stimulated experiment, and 102.0% (P<0.01), 54.9% (P<0.01), in ISO stimulated experiment, 5 and 10 min after Ang II or ISO insult, respectively, than the controls. IMD co-incubation with Ang II or ISO showed no any significant effect on the phosphorylation level of AKT kinases, while, augmented ERK1/2 phosphorylation, with 137.4% or 45.7% higher in phospho-ERK1/2/total ERK1/2 ratios after co-incubation of IMD with Ang II for 10 min, or IMD with ISO for 5 min, respectively, compared with Ang II or ISO alone group (both P<0.01). Taking the ability of IMD in induction of intracellular cAMP/PKA signaling into consideration, our results suggested that IMD augmented autophagy level in H9c2 cells would be mediated by cAMP-PKA and ERK1/2 signaling. To verify this hypothesis, we further investigated whether IMD prevented apoptosis by increasing autophagy in the presence of wortamannin (a specific PI3K inhibitor), H89 (a specific PKA inhibitor), or PD98059 (a specific MAPK/ERK1/2 inhibitor). Fig. 9B showed the intracellular signaling pathways triggered by IMD to stir a protective autophagy and blunt Ang II or ISO-induced apoptosis. Compared with the control without any inducer, 1 µM Ang II or 2 µM ISO incubation for 24 h significantly increased LC3-II/β-actin and cleaved caspase-3/β-actin ratios, and IMD (10^−**6**^ M) co-incubation further augmented LC3-II/β-actin ratios but reduced cleaved caspase-3/β-actin ratios, which suggested a strong negative correlation between IMD-induced autophagy and apoptosis in cardiomyocytes under hypertrophic stimuli. However, compared with co-incubation of IMD with Ang II or ISO group, pretreatment with the PI3K inhibitor wortamannin, PKA inhibitor H89 and MAPK/ERK1/2 inhibitor PD98059, and then co-incubated with both IMD and hypertrophic inducers, remarkably decreased LC3-II/LC3-I ratios, but only H89 and PD98059 co-incubation groups showed significant increase in cleaved caspase-3/β-actin ratios. In addition, no any statistical deference were observed in cell autophagy and apoptosis level between the control and IMD, wortamannin, H89,PD98059 or 3-MA alone group (all P>0.05, Figure 9A and B). Our results indicated that IMD inhibited cardiomyocyte apoptosis by induction of protective autophagy in cAMP/PKA- and MAPK-dependent intracellular mechanisms.

## Discussion

Cardiac cells can synthesize and release lots of local bioactive factors including catecholamines (CAs), natriuretic peptides (NPs) and calcitonin generelated peptide family members, especially adrenomedullin and intermedin [4]–[6]. Through activation of local autocrine and paracrine circuits, cardiovascular homeostasis in health and disease is regulated sophisticatedly [4]. Cardiac hypertrophy is an imbalance status in cardiac homeostasis, which is characterized by excessively activated local catecholamine and the rennin-angiotensin-aldosterone (RAS) systems, and these factors are required for the regulation of cardiac hypertrophy development in an autocrine/paracrine manner [4], [24]. As the important cardiac cellular compensatory response to the hypertrophic stimuli, a few protective factors are induced, which are orchestrated by sequential induction and/or release of cytokines resulting in a closely regulated cytokine cascade [4]–[6]. The upregulation of IMD peptide in pathological states has been proven to play an important protective role as an endogenous auto−/paracrine factor to antagonize organ damage [7], [8]. Bell and colleagues [25] have reported that the expression of endogenous IMD was robustly increased in hypertrophic hearts of hypertensive rats, which suggested a protective role of IMD in cardiac hypertrophy. Now, it is still unclear about the actions and underlying mechanisms of IMD in protection against cardiac hypertrophic diseases [7], [8].

In the present study, we observed that endogenous IMD expression was increased remarkably in mouse hearts treated by TAC and H9c2 cells stimulated with Ang II and ISO. These results were consistent with those reported by Bell and associates [25]. Furthermore, the mRNA expression of CRLR, RAMP1 and RAMP3 was augmented in hypertrophic cardiac tissues and H9c2 cells, which indicated that the special receptor complexes of IMD, CRLR/RAMP1 and CRLR/RAMP3 [9], were enhanced. The up-regulation of IMD-receptor system in hypertrophic cardiomyocytes hinted an important pathophysiological role of IMD in cardiac hypertrophic diseases. We further supplemented exogenous IMD, which has the same molecular structure and bioactivity as the wild type IMD peptide [15], to hypertrophic animals and cells, to reveal the regulation of IMD in cardiac hypertrophy. After the mice were induced into a pressure overloading condition by use of TAC for two and four weeks, the heart size, HW/BW, cross-sectional cardiomyocyte area, interstitial collagen, capillary density and hypertrophic biomarkers (ANP and BNP) expression were significantly increased, associating with the cardiac dysfunction. These results indicated that pressure overload induced serious cardiac remodeling, including myocardial hypertrophy, fibrosis and compensative capillary angiogenesis. The similar phenomenon was observed in 1 µM Ang II or 2 µM ISO-stimulated H9c2 cells, with remarkable increase in the cell size, ANP and BNP expression. In addition, cardiomycyte hypertrophy was paralleling with the significant increase in apoptosis in both mouse hearts and cell culture. IMD strongly protected cardiomyocytes against hypertrophy induced by pressure overload or hypertrophic stimuli in vivo and in vitro. We observed that IMD supplementation significantly decreased the heart size, HW/BW, cardiomyocyte size and apoptosis, interstitial collagen, ANP and BNP expression in hypertrophic mouse hearts and H9c2 cells, and markedly improved the cardiac functions and augmented the capillary density in cardiac tissues. Insufficient capillary density may cause impaired oxygen diffusion, myocyte necrosis and ultimately increased cardiac fibrosis [26]. These results indicated that IMD was not only effectively inhibited myocardial hypertrophic remodeling, but also hold a special feature to improve myocardial blood supply by enhancing angiogenesis. A few of mechanisms have been proposed for the cytoprotective effects of IMD in cardiovascular system, including enhancing cardiac contractile function via a protein kinase C- and protein kinase A-dependent pathway [27], and attenuating myocardial injury through a PI3 kinase/Akt/GSK-3beta signaling pathway [28]. Cardiac hypertrophy is usually associating with the activation in protein synthesis and dysfunction in protein degradation [1], [11], [29]. Autophagy is a highly conserved cellular mechanism of protein degradation and recycling, and plays an important role in the maintenance of cardiac homeostasis [11]. IMD has been proven to abolish protein synthesis in response to a hypertrophic stimulus [8]. However, little is known about its effects on protein degradation pathways [7], [8].

Autophagy serves both to recycle cellular components and to eliminate damaged proteins or organelles that might otherwise be toxic or trigger apoptotic death [12], [29]. Global loss of autophagy, as in the case of the Atg5-null mouse, results in cardiomyopathy and heart failure [11]. Cardiac-specific Atg5 or Atg7 deficiency leads to cardiac hypertrophy [11]. These founding reveals an essential protective role for autophagy in cardiac hypertrophy [11]–[13]. The amount of LC3-II correlates well with the number of autophagosomes [11], [29], and we used it as a reliable marker, together with GFP-LC3 fusion protein, to monitor autophagy. We observed that autophagy was remarkably increased in TAC-treated mouse hearts compared with the control, which is similar with the results reported by Zhou and colleagues [30] that increased autophagy contributed to a compensatory response to help cardiomyocyte survival by improvement of protein degradation [11], [12]. In H9c2 cells, we observed that compared with the controls, Ang II insult induced an increase in autopahgy level, while ISO exposure decreased autophagy activity till 12 h and then increased it. These results were compared with those reported by Porrello [31], Pfeifer [32] and their colleagues. Both TAC treatment and hypertrophic stimuli insults induced significant increase in cardiomyocyte apoptosis indicated by TUNEL assay and cleavage of caspase-3 protein level. IMD supplementation significantly decreased TAC or hypertrophic inducer-stirred apoptosis in hypertrophic cardiac tissues and H9c2 cells, associating with the increase in autophagy levels. We further evaluated the role of IMD-induced autophagy in cardiomyocyte apoptosis by the use of 3-MA, an autophagy inhibitor. Compared with Ang II or ISO insult alone groups, after 3-MA was used to decrease the IMD-induced autophagy level, the cardiomyocyte apoptosis was significantly increased. These results indicated that IMD induced a protective autophagy to help cardiomyocyte survival in hypertrophic conditions through an autophagy dependent manner.

To provide further biochemical evidence to reveal the underlying mechanisms of IMD in the induction of protective autophagy, we have investigated IMD’s intracellular signaling pathways leading to activation of autophagy and inhibition of hypertrophy-induced apoptosis. As an endogenous multi-functional counter-regulatory peptide in the cardiovascular system, IMD is proposed to activate the intracellular anti-apoptotic effects via the regulation of PKA, PI3K-Akt and mitogen-activated protein kinases (MAPKs) signaling pathways [7], [8]. We observed that IMD, Ang II or ISO incubation alone induced intracellular cAMP production, these results were similar to those reported by Pires [33], Thekkumkara [34], Uchida [35] and their associates, respectively. IMD supplementation remarkably augmented the cAMP contents in H9c2 cells stimulated by Ang II or ISO. PKA is also known as cAMP-dependent protein kinase, it is a family of enzymes whose activity is dependent on cellular levels of cAMP. Our results indicated that IMD-induced cAMP might cause an increase in PKA activity in H9c2 cells. We further observed the activation of ERK1/2 and Akt, measured as serine residue-specific phosphorylation. Both of them were induced by IMD, Ang II or ISO alone treatment, these results were comparable with those studies done by Smith [36], Das [37], Shizukuda [38] and their colleagues, respectively. Interestingly, compared with the Ang II or ISO alone group, IMD co-incubation with Ang II or ISO only increased the ERK1/2 phosphorylation, while showed no obvious effect on AKT activation. Our results indicated that the activation of IMD in both cAMP/PKA and ERK signaling pathway might contribute to its cardiac cytoprotection. Furthermore, we observed that PI3K inhibition by wortamannin, PKA inhibition by H89, or MAPK/ERK1/2 inhibition by PD98059 effectively reduced the IMD-augmented autophagy level in Ang II or ISO-exposed H9c2 cells, but only H89 and PD98059 pre-incubation abolish the anti-apoptotic action of IMD. These results indicated that IMD activated protective autophagy and attenuated apoptosis in hypertrophic cardiomyocytes via a cAMP/PKA and MAPK/ERK1/2 activation pathways.

**Figure 10:**
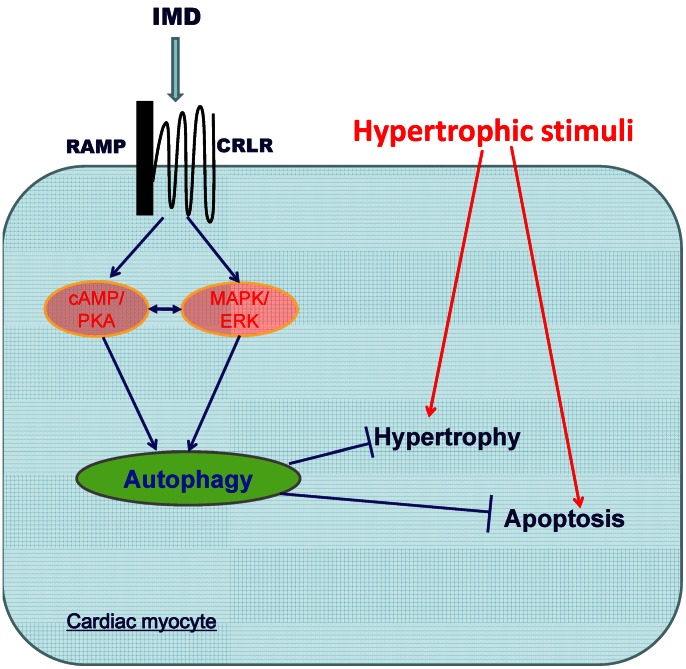
The schematic diagram summarized the molecular mechanisms by which IMD induced protective autophagy to suppress apoptosis in hypertrophic cardiomyocytes. In response to hypertrophic stimuli exposure, the hypertrophy is paralleling with the apoptosis in cardiomyocytes. IMD attenuates the hypertrophic stimuli-induced cardiomyocyte size increase and apoptosis by augmenting the protective autophagy level through the activation of cAMP/PKA and MAPK/ERK1/2 pathways. IMD, intermedin; CRLR, calcitonin receptor-like receptor; RAMP, receptor activity-modifying proteins.

In summary, as shown in Figure 10, the principal finding of this study was that the autophagy stirred by IMD supplementation is involved in its protection against cardiomyocyte hypertrophy and apoptosis induced by pressure overload and hypertrophic stimuli through the activation of both cAMP/PKA and MAPK/ERK1/2 pathways.
